# Extended reality technologies in small and medium-sized European industrial companies: level of awareness, diffusion and enablers of adoption

**DOI:** 10.1007/s10055-022-00662-2

**Published:** 2022-06-29

**Authors:** Henri Jalo, Henri Pirkkalainen, Osku Torro, Elena Pessot, Andrea Zangiacomi, Aleksei Tepljakov

**Affiliations:** 1 Tampere University, Tampere, Finland; 2 National Research Council of Italy, Milan, Italy; 3 Tallinn University of Technology, Tallinn, Estonia

**Keywords:** Augmented reality, Virtual reality, Extended reality, Technology adoption, Industry 4.0, Small and medium-sized enterprises

## Abstract

Augmented reality (AR) and virtual reality (VR), collectively referred to as “extended reality” (XR), have begun to diffuse in industry. However, the current levels of awareness, perceived limitations, and use of AR and VR, as well as the potential differences on these aspects between these technologies are still not well known. Moreover, it is unknown whether small and medium-sized enterprises (SMEs) differ from large companies on these issues. This research employed a mixed methods research design to address this gap by carrying out a cross-sectional survey (*n* = 208) to gauge European industrial companies’ level of AR and VR awareness and adoption, and by interviewing 45 companies in nine European countries in order to identify critical enabling factors in the adoption of XR for SMEs. Results show no statistical difference between the respondents’ perceptions toward AR and VR or in their use levels. Thus, examining AR and VR under the umbrella term XR seems justified, especially in the context of their organizational use. However, larger companies were found to be using XR more than SMEs. Analysis of interviews based on the technology–organization–environment framework also yielded several enabling factors affecting XR adoption and specified whether they are particularly highlighted in the SME context. Overall, this paper contributes to XR research by providing a holistic multi-country overview that highlights key issues for managers aiming to invest in these technologies, as well as critical organizational perspectives to be considered by scholars.

## Introduction

In recent years, the augmented reality (AR) and virtual reality (VR), collectively known as extended reality (XR), markets have been predicted to grow significantly (e.g., Grand View Research 2021; IDC 2020). However, these predictions usually pertain to all types of use, both consumer and enterprise. Accordingly, the current level of XR use in organizations, and, more specifically, in small and medium-sized enterprises (SMEs), is still not clear, and further investigation is needed. Due to the slowness of diffusion in consumer use, many XR developers have also begun pivoting toward the enterprise sector. For example, Google Glass and Magic Leap were initially aimed at the consumer sector, but later began focusing their offerings toward enterprise clients (Hammond 2020; Miller 2015). Moreover, many of the major technology companies are now developing XR solutions specifically for enterprise use (e.g., Microsoft Mesh and Nvidia Omniverse).

Accordingly, both AR and VR have been identified as technologies that organizations could potentially capitalize on (see e.g., Berg and Vance 2017; Porter and Heppelmann 2017; Torro et al. 2021); this fits as part of the overall trend of digitalization, which has been critical for the competitiveness of companies during recent decades. Companies’ interest in utilizing XR has also increased consistently, especially among larger companies (Porter and Heppelmann 2017). However, SMEs have been lagging behind larger companies when it comes to digital transformation (OECD 2021), due to lack of resources and more focused competencies, which affect their innovation capability and readiness to digitalize their operations (Denicolai et al. 2021). As the pace at which technology is adopted increases (Denning and Lewis 2020), SMEs risk being left behind. This can have dire consequences for societies as SMEs constitute the majority of all businesses and employ the majority of people (European Central Bank 2021). It is therefore crucial to understand the overall organizational situation and the challenges that accompany XR in order to support companies in their adoption efforts.

Interest in XR is also growing from the research viewpoint. Nevertheless, most of the extant organizational XR studies have focused on a single industry or country; very few overall quantitative accounts exist on the topic of how widespread their use is. Due to the rapid developments in XR, it is important to assess how much these technologies have diffused into enterprise use and what issues companies perceive to be critical in their adoption. This paper aims to examine the overall diffusion of XR in European industrial companies as of 2020, with a specific focus on SMEs. The twofold research question of this paper is:

1. Do the current levels of XR awareness, use, and perceived limitations differ between European SMEs and larger companies?
2. What are the critical enabling factors of XR adoption for SMEs?

We employed a mixed methods research approach to get a wider and more complete perspective on XR adoption (Venkatesh et al. 2013). Firstly, quantitative data were collected via a cross-sectional online survey with 208 respondents from European companies belonging to different sectors. The analysis examined the potential differences in perceptions toward AR and VR and investigated whether it is empirically justified to examine AR and VR conjointly under the recently popularized umbrella term XR. In addition, 45 semi-structured interviews were carried out in nine European countries to explore XR adoption in more depth. The interviews were framed and analyzed by using the Technology*–*Organization*–*Environment (TOE) framework (DePietro et al. 1990). The analysis identified relevant XR adoption enabling factors for companies and evaluated whether the enabling factors were specifically highlighted in the SME context.

This paper contributes to research by providing a comprehensive overview about the current level of AR and VR adoption and use in European industrial companies. We found no statistical difference in the respondents’ perceptions or level of use between these technologies. However, AR and VR use levels were found to differ between SMEs and large companies, although the levels of awareness and perceived limitations of adoption were similar for both types of companies. We also uncovered 13 important enabling factors affecting XR adoption, eight of which were noted to be especially important in the SME context. This contribution is valuable, as these technologies have had several previous waves which failed to materialize into widespread industrial use (Walsh and Pawlowski 2002). These findings help to illuminate the most important determining factors affecting their adoption that organizations (especially SMEs) would benefit from investigating.

The rest of the paper is structured as follows. First, the theoretical background on AR and VR as well as their adoption is examined in Sect. 2. Second, the methodology relating to the survey and semi-structured interviews is described in Sect. 3. Third, the results of the survey and the findings from the interviews are presented in Sects. 4 and 5. Finally, the results and findings are discussed in Sect. 6, along with the contributions and limitations of the study. The paper ends with suggestions for future research.

## Literature review

AR can be defined as a technology that *combines or superimposes digital information into the user’s view of the real world* (Azuma 1997), and VR as a technology that *replaces the user’s view of the real world with an immersive and interactive 3D virtual environment* (Bryson 1995; Jerald 2015). Both AR and VR utilize head-mounted displays (HMDs) to achieve these outcomes for the user; however, smartphones and tablets are also widely used to create AR experiences (Jalo et al. 2020; Porter and Heppelmann 2017). AR and VR can essentially be seen as tools with which one can present digital information to the user in a more immersive and interactive fashion (Davila Delgado et al. 2020). This is achieved either by transplanting information into the real-life context with AR, or by examining it in a completely virtual space in VR. AR and VR are also often researched in conjunction (see e.g., Cipresso et al. 2018; Li et al. 2018; Ong and Nee 2013; Steffen et al. 2019), and, more recently, the umbrella term XR has been used to refer to both AR and VR together (Bujić et al. 2021; Chuah 2019; Dwivedi et al. 2021; Gong et al. 2021). However, it is uncertain if the use levels and perceptions of industrial companies differ between these technologies, or whether common adoption factors affect their implementation in the organizational context.

Recent years have seen an increase in empirical research on industry adoption of XR from the organizational point of view. Ten articles examining organizational XR adoption were identified in total for this literature review. Three of these articles examined XR adoption with a cross-industry sample (Berg and Vance 2017; Masood and Egger 2019, 2020). Berg and Vance (2017) qualitatively surveyed the use of VR in the US; they found measuring the return on investment (ROI) of VR to be important for maintaining top management support. Tailoring the VR solution to focus either on visual fidelity or interactivity and technical details depending on the user group was found to be an important factor for leveraging the affordances of VR for specific use cases. Masood and Egger (2019) conducted a survey on the importance of various adoption factors for AR. They found organizational fit, technology compatibility and hardware maturity, and tailoring of the system to fit the organization’s needs via piloting and user training to be statistically significant factors affecting AR adoption. Masood and Egger (2020) further examined AR adoption factors based on practical field experiments using AR HMDs with organizations from the UK and found user acceptance to be crucial for AR adoption; the study also confirmed the importance of system configuration and organizational fit.

Five articles examining XR adoption in the architecture, engineering, and construction (AEC) context were also identified (Badamasi et al. 2022; Davila Delgado et al. 2020; Jalo et al. 2018, 2020; Noghabaei et al. 2020). Jalo et al. (2018) identified different factors affecting the adoption of AR in the Finnish Facility Management industry via interviews and focus groups. They found the compatibility of information systems (IS) and AR and wider interorganizational cooperation to be crucial for their organizational use. Jalo et al. (2020) researched different enabling factors relating to social virtual reality (SVR) diffusion in AEC organizations in Finland via interviews and focus groups. They found that identifying visual work tasks that rely on remote collaboration leads to an increase in adoption. IS and software compatibility with SVR and ensuring multi-device access to SVR were also found to aid in SVR diffusion. Lastly, the perceived complexity of SVR could be mitigated by utilizing more user-friendly stand-alone VR HMDs, as well as by training users in 3D modeling skills, by carrying out initial testing in a group, and by designating VR lead users. A survey study conducted in the UK by Badamasi et al. (2022) found that the high cost of VR devices, employees’ lack of VR skills, and the required cultural change brought about by VR adoption were the most crucial barriers hindering the adoption of VR. Davila Delgado et al. (2020) identified relevant XR adoption factors in the UK via focus groups and ranked them based on a quantitative survey. Similar to Badamasi et al. (2022), high costs and low maturity of XR technologies, lack of XR skills, and general reluctance regarding new technologies were identified as the most critical limiting factors in their study. Lastly, Noghabaei et al. (2020) carried out a two-wave survey in the USA and found lack of financial resources and lack of knowledge about XR within top management and design teams to be the most important barriers for XR adoption.

The two final articles examined XR adoption in the retail context (Chandra and Kumar 2018; Alam et al. 2021). Chandra and Kumar (2018) examined the adoption of AR in e-commerce in Singapore, India, and the USA with a survey. Their results highlighted the key roles of relative advantage, securing top management support, the readiness of customers to use AR, and a sufficient level of technological competence to implement and maintain AR in increasing organizational adoption intention. Alam et al. (2021) used a survey to assess which factors influence the adoption of AR in Malaysian retail companies. They found that pressure from competitors and customers, and the managers’ technological knowledge and awareness of AR to be key drivers of AR adoption, whereas high costs relating to AR were hindering adoption. Moreover, the perceived usefulness of AR and the managers’ self-efficacy influenced managerial attitudes and adoption intentions.

The literature review shows that this stream of research has largely focused on a single country or industry, so studies containing larger multi-country and multi-industry samples could enhance the transferability and generalizability of the findings for industry in general. Moreover, key issues for SMEs are often not considered. Our study thus aims to provide a more holistic perspective on the current stage of XR adoption in industry, as well as to identify which adoption enablers are specifically critical for SMEs.

## Methodology

This study was carried out according to a mixed methods approach (Venkatesh et al. 2013), including a cross-sectional online survey and semi-structured interviews. Both the survey and interviews were carried out between April 2020 and October 2020. This research had a specific focus on SMEs due to their prominent role in the European manufacturing industry, and the related need to understand the current gaps in SMEs’ innovation processes in order to properly understand and support the implementation of new digital technologies such as AR and VR. However, larger companies were also included in the sample to provide a more complete and possibly contrasting view on the adoption of AR and VR. First, possible differences in the companies’ situations and perceptions between AR and VR were investigated and SMEs’ and large companies’ situations with these technologies were compared via the survey. Second, a set of relevant adoption factors relating to both technologies emerged from the semi-structured interviews. Their importance for SMEs was then evaluated and corroborating evidence for the enabling factors was sought from the extant literature.

The online survey statements were exploratory in nature and were formulated to address the respondent companies’ awareness and perceived limitations pertaining to AR and VR and their current level of use. In the survey, each respondent answered identical questions related to AR and VR according to a 7-point Likert scale ranging from “strongly disagree” to “strongly agree,” or a 5-point Likert scale ranging from “never” to “a great deal,” based on the specific question. The statements are listed in Tables 2 and 3. All of the statements were first asked about AR, followed by VR. The order of the statements was not randomized, however, as the identical questions were not placed directly after each other, the respondents were less likely to be induced to answer them similarly (Nederhoff 1985). The survey was revised based on feedback from pilot tests with two SMEs in Finland and Italy. The final survey instrument was then translated into German, Italian, and Spanish. The survey included questions about the background of the respondents, followed by questions about the overall status of AR and VR use in their companies.

The survey was carried out in the context of a European research project and distributed among the professional networks of the research consortium. As we had no means to secure responses from each potential respondent, we acknowledge the possibility of some degree of selection bias, as those who are already familiar with AR or VR to some degree or are interested in these technologies are possibly more likely to answer such a survey (Armstrong and Overton 1977). However, based on the results of the survey, this bias is likely not significant, with approximately 60% of the respondents still answering that their companies do not use AR or VR. We also aimed to reduce nonresponsiveness by assuring the respondents that their anonymity would be protected (Armstrong and Overton 1977). The survey was opened by 451 people; 208 people provided valid complete responses to the survey (159 of these were from SMEs). We thus had a response rate of 46.1%, which is consistent with prior IS adoption studies (see e.g., Kim and Kankanhalli 2009; Wolf et al. 2012). We also assessed nonresponse bias by carrying out a Levene’s homogeneity of variance test with respondents from the first and last 33% of the responses to the statements in Tables 2 and 3 (Armstrong and Overton 1977). The early and late groups did not differ from each other in a statistically significant way (*p* > 0.05 for all group comparisons), suggesting that nonresponse bias was not a significant threat to the results.

The survey respondents were quite evenly distributed between lower (24%), middle (31.3%), and top management (30.8%), with 13.9% choosing the option “other” (indicating they were experts or other employees). A clear majority (79.8%) of the respondents were male and the rest (20.2%) were female. As for the age of the respondents, 31.2% of them were 18–34 years old, 52.8% were 35–54 years old, and 16% were 55–74 years old. A majority of the respondents (61.5%) had an advanced degree (Master's, Ph.D., M.D.), 22.6% had a bachelor’s degree, and the rest (15.9%) had a lower level of education (e.g., a high school degree).

Table 1 provides background information about the companies of the respondents. As shown in Table 1, no single industry or country dominated the responses. Most of the responses (76.5%) were from SMEs (with under 250 employees), with the remaining responses coming from larger companies. Most of the companies were also internationally focused (58.5%) or operated at least at the national level (29.3%). The companies were also mainly focused on providing products and solutions to other companies (60.6%) or consumers (16.3%).

**Table 1:** Information about the respondents’ companies (*n* = 208)

	Frequency and percentage		Frequency and percentage
*Location*		*Industry*	
Austria	24 (11.5%)	Aerospace	5 (2.4%)
Belgium	25 (12.0%)	Architecture and construction	32 (15.4%)
Cyprus	27 (13.0%)	Automotives and vehicles	17 (8.2%)
Estonia	11 (5.3%)	Biotechnology	1 (0.5%)
Finland	28 (13.5%)	Chemicals	3 (1.4%)
Germany	20 (9.6%)	Clothes and textiles	1 (0.5%)
Greece	2 (1.0%)	Computers and electronics	14 (6.7%)
Ireland	1 (0.5%)	Electrical equipment	6 (2.9%)
Italy	16 (7.7%)	Food and beverages	8 (3.9%)
Netherlands	24 (11.5%)	Furniture	3 (1.4%)
Romania	1 (0.5%)	Healthcare and pharmaceuticals	14 (6.7%)
Spain	29 (13.9%)	Industrial installation and maintenance	12 (5.8%)
*Employees*		Machinery and equipment	21 (10.1%)
1–9	39 (18.8%)	Metals	19 (9.1%)
10–49	66 (31.7%)	Plastics	3 (1.4%)
50–250	54 (26.0%)	Other (e.g., Consulting)	49 (23.6%)
251–500	13 (6.2%)		
501–1000	5 (2.4%)		
> 1000	31 (14.9%)		

IBM SPSS Statistics version 27 was used to perform the data analysis. The survey data was first tested for normality with the Shapiro–Wilk test, which indicated that all of the data were non-normally distributed (*p* < 0.001). After the data were logarithmically transformed, they were still found to be not normally distributed. Therefore, the nonparametric Wilcoxon Signed-Rank (WSR) and Mann–Whitney U tests were adopted to carry out the analysis, as recommended in the literature (Blair and Higgins 1985; Rasmussen and Dunlap 1991; Serlin and Harwell 2004). The WSR test was used to compare the differences between AR and VR within the entire sample (*n* = 208). A within-subjects repeated measures design was thus used for this test. We also carried out a Spearman’s nonparametric correlation test on the answer pairs. The differences between the SMEs (*n* = 159) and large companies (*n* = 49) were examined with the Mann–Whitney U test, which can be used to compare two independent samples with different sample sizes (George and Mallery 2019).

The semi-structured interviews were also carried out as part of the activities of the same European research project involving several researchers from different countries, with the lead author providing the interview protocol for the other researchers. The interviews represented multiple case-studies involving 45 companies from nine European countries (five from Austria, Belgium, Cyprus, Estonia, Finland, the Netherlands, and Spain, six from Italy, and four from Germany). Of these companies, 31 were SMEs (below 250 employees) and 14 were large companies (over 250 employees). Interviewees consisted of senior management (16), middle management (18), lower management (4), and experts (7). The interviewees were selected by the project partners from their professional networks. Purposeful sampling was thus used at this stage to gather insights from companies that were either considering using XR or had already adopted such solutions (Patton 2002). The information collected through these interviews was also integrated with secondary sources, including internal documentation provided by companies, as well as data available on the internet (e.g., company websites), in order to triangulate data and assure the consistency of related findings (Yin 2013).

In order to provide a comprehensive accounting of factors affecting the adoption of XR in organizations, the TOE framework was utilized in structuring the interviews and in analyzing the collected data to illuminate relevant technological, organizational, and environmental adoption factors (DePietro et al. 1990). The TOE framework provided a good basis for further identification of enabling factors from the data, as it does not predetermine the particular factors influencing adoption. The application of the TOE framework has also found wide empirical support in the context of many Industry 4.0 technologies (e.g., Borgman et al. 2013; Chandra and Kumar 2018; Martins et al. 2016), and it has been argued to be useful for analyzing the adoption of novel technologies in the context of a wide variety of organizations (Schiavone et al. 2022), attesting to its suitability in our research context. The interview protocol (see “Appendix”) was developed based on the themes highlighted in the literature review described in Sect. 2 and the lead author’s experience on several XR research projects. The interview protocol was also circulated among the researchers and refined based on their feedback. The interviews were carried out via remote video conferencing software (such as Zoom and Microsoft Teams) due to the COVID-19 pandemic. The interviews lasted between 45 and 75 min and were recorded with the consent of the interviewees. The semi-structured nature of the interviews allowed the interviewers flexibility to ask follow-up questions and follow the natural flow of the conversation, while still following a common structure (Gillham 2005). The interviewers created a summary of all the interviews and transcribed insightful quotes from each interview into English. The qualitative analysis of the interviews adopted an interpretive approach (Walsham 1995). The analysis process began by giving codes to smaller pieces of data, and then grouping them under the TOE framework categories (Creswell 2015; DePietro et al. 1990). These codes were iteratively combined into higher-level main codes to develop themes (Creswell 2015). Disconfirming evidence for the themes was also sought from the data (Creswell and Miller 2000). Illustrative quotations for each main code are included in the findings. The coding was mainly done by the lead author; however, co-authors later reviewed the findings to confirm their accuracy, as they also participated in the data collection (Creswell 2015).

Next, we will first present the analysis of the survey data, followed by the findings from the interviews.

## Companies’ perceptions and adoption levels of AR and VR

The WSR test (Table 4) was carried out on the survey data to determine if there were statistical differences between the answers to similar statements in terms of awareness, limitations, and use of AR and VR (depicted in Tables 2 and 3). Overall, if the companies were to perceive these technologies to be very different, we would expect the distribution of the responses to change between AR and VR statements.

**Table 2:** Survey responses on AR and VR awareness and limitations (*n* = 208)

	Strongly disagree	Disagree	Somewhat disagree	Neither agree nor disagree	Somewhat agree	Agree	Strongly agree
Our organization is well-aware of the potential of AR [1]	18 (8.7%)	33 (15.9%)	15 (7.2%)	18 (8.7%)	47 (22.6%)	39 (18.8%)	38 (18.3%)
Our organization is well-aware of the potential of VR [1]	17 (8.2%)	31 (14.9%)	16 (7.7%)	20 (9.6%)	42 (20.2%)	43 (20.7%)	39 (18.8%)
There are many limitations to using AR in our organization. [2]	17 (8.2%)	34 (16.3%)	21 (10.1%)	53 (25.5%)	40 (19.2%)	31 (14.9%)	12 (5.8%)
There are many limitations to using VR in our organization. [2]	16 (7.7%)	37 (17.8%)	23 (11.1%)	43 (20.7%)	38 (18.3%)	37 (17.8%)	14 (6.7%)

**Table 3:** Survey responses on AR and VR use levels (*n* = 208)

	Never	Rarely	Occasionally	A moderate amount	A great deal
Our organization is making use of AR [3]	123 (59.1%)	27 (13.0%)	30 (14.4%)	16 (7.7%)	12 (5.8%)
Our organization is making use of VR [3]	119 (57.2%)	24 (11.5%)	25 (12.0%)	21 (10.1%)	19 (9.1%)

In the WSR test, if the significance levels of the paired samples reach statistical significance (*p* < 0.05), the test would indicate that there is a statistically significant difference in the medians between the two samples. From Table 4, we can see that none of the pairs reach this level of significance. We can therefore conclude that there was not a statistically significant difference between AR and VR perceptions or use for the respondents in this sample. The correlations between the answer pairs were also significant at the *p* < 0.01 level, with the first pair having a strong positive correlation and the last two having a moderately strong positive correlation (Dancey and Reidy 2007). The respondents’ answers were thus significantly paired in regard to AR and VR (i.e., if they were well aware of the potential of AR, they answered similarly with regards to VR). These analyses provide further evidence justifying examining AR and VR conjointly as XR, as has already been done in previous literature (e.g., Davila Delgado et al. 2020; Steffen et al. 2019). Thus, the following analysis in Sect. 5 will also examine these technologies collectively as XR.

**Table 4:** Wilcoxon signed-rank test and Spearman’s correlation test results (*n* = 208)

	*Z*	*p*	Spearman’s correlation
Our organization is well-aware of the potential of [AR/VR]	− 0.724	0.469	0.745**
There are many limitations to using [AR/VR] in our organization	− 0.447	0.655	0.652**
Our organization is making use of [AR/VR]	− 1.449	0.147	0.455**

**p < 0.01

Finally, we also tested for differences between SMEs and large companies within the sample with the Mann–Whitney U test. As can be seen in Table 5, SMEs and large companies differed significantly in their AR use (*p* = 0.011, *p* < 0.05) with a small effect size (*r* = 0.175) and statistically more marginally with VR use (*p* = 0.06, *p* < 0.1) with a small effect size (*r* = 0.13; Cohen 1988). However, there were no differences in their awareness levels or perceived limitations to using these technologies.

**Table 5:** Mann–Whitney U test results comparing SMEs (*n* = 159) and large companies (*n* = 49)

	Median (SME)	Median (Large)	*U*	*Z*	*p*
Our organization is well-aware of the potential of AR	5	5	3853	− 0.117	0.907
Our organization is well-aware of the potential of VR	5	5	3734	− 0.445	0.656
There are many limitations to using AR in our organization	4	4	3578.5	− 0.875	0.381
There are many limitations to using VR in our organization	4	5	3594.5	− 0.829	0.407
Our organization is making use of AR	1	2	3068.5	− 2.530	0.011
Our organization is making use of VR	1	2	3272.5	− 1.882	0.060

## XR adoption enabling factors

In this section, the qualitative findings of the study are described by distinguishing technological, organizational, and environmental enabling factors for XR adoption, based on the TOE framework.

### Technological factors

Main technological enabling factors that emerged from the findings include: the extent of the XR hardware install-base and related network effects, finding the right balance of features in XR hardware (depending on the business process), securing XR testing opportunities, and ensuring XR and IS compatibility as well as rapidness of IS-XR workflows.

#### Technological install-base and network effects

Many of the interviewees noted that the required install-base for widespread XR use was often missing. For instance, one interviewee noted that their customers do not have VR HMDs which could be used in business processes. If XR hardware are still not widely diffused, the network effects which can induce others to adopt XR are also lower. Specialized XR applications are also often unavailable for various industry contexts.

“I had great hope that somebody in the VR community would have provided apps for our profession to improve safety at work. I know some apps for [our] industry, but unfortunately, they are not suitable for our needs.” Development Manager, Austria

Overall, companies show a higher readiness in the AR context as AR can often be utilized with existing smartphones with both internal and external stakeholders. Nevertheless, smartphones with the required features for advanced AR solutions are still not widely in use in industry. However, this issue was seen to automatically improve over time as stakeholders switch to newer devices.

#### Balancing performance and ease of use in XR hardware

The companies widely noted the challenge of finding the correct balance between visual fidelity, performance, ease of use, and quick setup of the XR devices. For VR, stand-alone HMDs were widely seen to be the preferred option due to their simplicity and smooth user experience, which were seen to be especially important factors in customer-facing business processes. However, tethered VR HMDs were still preferred in use cases that require more advanced functionalities and higher visual fidelity (e.g., high-end presentations).

“I see that there’s a divide [on what type of VR will be used]. For example, the design cases, work site, and design meetings will use stand-alone [VR] because they need to be as easy to use as possible. The cost is also an issue […]. Then again, if we want to sell something specific to clients, in that case it tilts toward the higher quality [VR] glasses.” Manager, Finland

For AR, the interviewed companies had mainly focused on using smartphones and tablets (hand-held devices, or HHDs); HMDs (e.g., Microsoft HoloLens) were preferred in tasks where freedom of movement for both hands was needed. However, some interviewees noted that AR HMDs were still often not robust enough for industrial use, especially in more demanding conditions (e.g., dust and rain). In the short term, HHDs were seen to offer the most potential due to their wide install-base, low costs, and minimal training needs. The AR capabilities of these devices were also expected to increase automatically over the years; however, more advanced AR use cases (e.g., fitting wiring schematics in a building site) still had accuracy and reliability challenges.

“We have to be sure about the reliability and precision of these technologies before their use. Is the presented information and data accurate? There is no room for big mistakes in construction. Small mistakes can cost a lot of money.” Manager, Cyprus

#### Opportunities to test XR devices and software

Many of the interviewed companies had been testing a wide variety of XR devices. Practical opportunities to test the XR devices were seen as one of the crucial enablers to understand their potential and challenges. The main limitation here was seen to be with hardware, as, on the software end of things, most enterprise XR solutions in fact frequently offer free trial periods. Due to their novelty, relatively high cost, and initial complexity, many interviewees found that facilitated and supportive settings (such as industry and university events) provided the best opportunities for experimenting with the newest solutions. However, in particular the interviewed SMEs were often unaware of these possibilities. A crucial limitation that was widely reported in the testing situations was that they did not enable multi-user testing, a feature that is judged to be fundamental to using these tools collaboratively.

“We would like to be offered a demonstration or a free trial before we use these technologies, in order to be sure about the results and if it actually produces profit for the company.” Manager, Cyprus

#### XR compatibility with information systems and software

Recent improvements in XR hardware were seen to be essential for their usefulness, however, their compatibility with organizational IS was still a barrier for their widespread adoption. Consequently, many interviewees had decided to first focus on modernizing their IS to enable later XR adoption and to ensure that application programming interfaces (APIs) were available for easy data access. Some interviewees also noted the difficulty of integrating legacy assets into XR processes (such as 2D design drawings), and thus judged the path-dependence from earlier choices with organizational IS to be a key limitation. Due to the challenges and work required in this area, especially with highly customized IS, many interviewees expected that many legacy assets would remain siloed and would not be incorporated into XR environments. For instance, the AEC industry has been transitioning toward using digital design tools (namely building information modeling, BIM), but very little digital information was reported to exist for many of the older properties. Accordingly, an AR maintenance app, for instance, could thus only be used in the context of new buildings.

“The issue with this technology [XR] is the lack of compatibility and integration with current systems and CAD software.” Business Operations Manager, Germany

#### Fast workflows between information systems and XR

Many interviewees noted that they had experienced significant difficulties with the speed of workflows between XR and their existing IS. As an example of the importance of this factor, the CEO of one of the interviewed companies reportedly changed his mind completely about VR after he saw the design information being transferred quickly between their design software and a VR software. This was seen to be crucial for the practicality and efficiency of new VR-enabled business processes. Some interviewees also reported that digital content can often already be accessed in AR or VR from the software with a single click, and that the cumbersome and time-consuming file transformations between several different software were not needed anymore. Automatic bi-directional workflows from software and IS to XR (and back) were seen as a key enabler in reducing work redundancy and in ensuring the reliability of the decisions and work being done in XR.

“Historically the workflows have been more custom [for VR], so we’ve exported the model into something else, then something more was done to it in some other software, and only then it became viewable, and even then not necessarily in a multi-user setting. Whereas now when we have the model, there’s a button which says ‘View in VR,’ and we can then go view it with a group.” Manager, Finland

### Organizational factors

Five organizational enabling factors were identified, including: securing top management support via practical XR testing, availability of XR development resources, ability to recruit XR experts, mitigating potential employee resistance toward XR, and effective facilitation of the initial XR adoption and use situations.

#### Top management knowledge and first-hand experience with XR

In many of the interviewed companies, the top management was generally aware of the potential of XR, but most companies had not yet actively begun implementing it. Top management interest and willingness to promote XR in the company was thus seen as a critical enabler. Practical experience and testing of XR devices and software by top management was seen to help them better understand their applicability and limitations in their company and thus secure the needed adoption resources. However, finding the time for upper management to learn how XR could transform organizational business processes was still a limitation.

“The CEO’s view on these technologies has become much more positive, he’s really taken this whole development thing as his own. He’s been complaining that we’ve been talking about this for years and years, (I’ve been working here for a year now), and we’ve just refined these things further but haven’t gotten to the practical part yet. Now it’s much more like ‘Let’s take this app into use,’; ‘Show this to the people at the building site and ask them whether this could be a good thing.’” Manager, Finland

#### Availability of resources and personnel for XR research and development

The perceived complexity of XR adoption was seen to require that key employees familiarize themselves with XR in detail and evaluate its effects on the company’s business processes, or even the overall business model. Most of the interviewed companies reported struggling with this issue and found it to be a limiting factor in adopting XR or expanding its use. In particular, SMEs felt this to be a key challenge as they reported already being stretched thin on personnel; however, larger companies did not feel this to be as serious of an issue.

“The pitfall however is that management doesn’t free up time for the employees to delve deeper into this technology and to do some experiments. As a consequence, only the most basic features of the software are used and the other features remain unexplored.” Manufacturing Engineer, Belgium

#### Ability to recruit people with XR expertise

Another challenge faced by the interviewed companies was in finding employees with XR experience. In the short term, XR competences were seen to be achieved either by self-learning or at university courses. One interviewee also noted that many of the employees with XR skills would likely not have extensive industry experience and transforming the company’s business processes with XR would thus need to be done in cooperation with senior employees.

“One of the problems we have is that our age distribution is such that we have guys like me [younger generation] and then there are supervisors that are closer to 60. To get them to use it [XR], we have to balance for a while between two things; I handle the facilitating [relating to the use of XR], and the other guy handles the construction management side [...], and then we try to share [domain] knowledge between us, because we still don’t have people who can handle both.” Manager, Finland

Many of the interviewees also noted that they were still more familiar with XR in entertainment rather than industrial use. Accordingly, some interviewees noted that these hedonic experiences could be used as a good starting point for thinking about how XR could be used in their companies.

#### Mitigating employee resistance toward adoption

Employee resistance toward adopting XR, especially from older employees, was identified as a crucial barrier. As XR can be used to transform operations significantly (e.g., from physical design reviews to remote XR reviews), the readiness and proclivity from both the management and the employees to adopt new ways of working was seen to be essential. An organizational culture that supports innovation and testing of technologies with a low threshold were seen to be important for mitigating possible user resistance. Incorporating employees into the XR adoption process from the beginning was also seen as a one of the greatest potential mitigation strategies; as one interviewee explained:

“Older employees were a bit skeptical about this technology [AR], but they have been consulted from the start, resulting in two equivalent systems they can choose from (Vuzix glasses or tablet) and finally, the whole technological change has been accepted and turns out to be successful today.” COO, Belgium

Providing extra hands-on training both for XR use and other enabling technologies (e.g., digital model exporting from IS) were seen to be essential in ensuring a smooth adoption process. The employees were seen to need sufficient XR skills to operate the solutions independently in order to transfer the ownership for the solutions to the business units. Although support should be available when needed, the primary responsibility for using the XR solutions effectively should be with the end-users.

#### Facilitating the initial adoption and use

Due to the importance of overcoming the initial skepticism and inertia toward XR adoption, some interviewees noted the importance of designing the first XR testing and adoption events to be as practical and engaging as possible. These sessions should include hands-on testing of devices as well as identification of a few key users who would be trained to be able to provide peer support.

“[...] it’s the job of sales to train the sellers to use VR, I’ll certainly be there to support as well, or I’ll train the main users who will then take it into everyday use so it’ll come into use more effectively. Then the know-how and ownership are there too.” Manager, Finland

Expectation management was also noted to be important due to the existing misinformation about XR caused by the hype surrounding these technologies. Choosing multi-user XR solutions with advanced user management features (e.g., gathering users to the event manager) was also identified to be important to ensuring smooth initial XR experiences. These solutions should also be multi-device compatible in order to enable reluctant users to participate in testing by viewing the XR event via, for example, desktops.

### Environmental factors

Three environmental enabling factors were identified: XR capabilities and readiness of the company’s stakeholders, competitor pressure from successful XR use, and the maturity of the XR vendor and training ecosystem.

#### Increased stakeholder XR capabilities and readiness

To enable large-scale XR use, many interviewees emphasized that their stakeholders need to increase their skills and readiness to use XR. Companies operating globally faced the largest limitations in this regard, because the significant heterogeneity of their stakeholders’ XR capabilities constrained the use of XR to a few select partners. This meant that XR could currently mainly be used in internal operations or in facilitated settings with customers.

“I think the most needed skill would be to train our customers in using AR technology to report missing or damaged machinery parts and to order replacements. This is also one of the reasons why the use of AR in our customer service is currently not considered economically viable. […] The main problem with this is that our clients are located all over the world, sometimes in very remote places. Adopting AR technology does not happen overnight and requires some basic infrastructural elements.” Clerk, Austria

Some interviewees also noted that their customers still often preferred to use smartphones or tablets rather than HMDs. Cultural factors were also seen to have a role in determining stakeholders’ propensity toward XR use.

“Especially in Italy the customer prefers to see the service person face-to-face, physical meetings are still preferred to solve problems.” Vice President, Italy

#### Observed XR benefits achieved by competitors

Most of the interviewees reported that their competitors and other relevant stakeholders were still not using XR in a significant way. Many interviewees noted that once XR use starts becoming more widespread, companies would start feeling the pressure to adopt XR solutions. However, it was seen to be easier for companies to identify competitors using XR in customer-facing business processes rather than in internal operations. Moreover, many interviewees noted that SMEs often wait for larger companies to successfully adopt and thereby demonstrate new technologies’ applicability before they consider adopting it. This risk-aversion was mostly due to their limited resources when compared to larger companies.

“Extended use of these technologies by competitors or relevant partners can influence our company to adopt them.” Manager, Cyprus

#### Maturity of XR vendor and training ecosystem

Many of the interviewed companies noted that they do not have many employees who would have the technological inclination to delve into XR to find out what solutions would work best for them**.** Generally, most of the information technology (IT) infrastructure and maintenance is outsourced in SMEs, decreasing SMEs’ internal capabilities to adopt and integrate new technologies. In contrast, some of the larger interviewed companies felt they would be able to adopt XR independently. Many of the interviewees thus felt it was essential for them to identify a suitable external partner or vendor who could handle the required XR hardware and software installations.

“We would need to purchase full equipment (hardware and software) and we need external consulting in order to know which equipment is best for our needs and purposes.” IT and HR Manager, Austria

Overall, there were significant differences between the companies in their abilities to adopt XR independently. For example, one interviewee noted that he could most likely carry out the installations independently because he had already spent a lot of time learning about XR devices, software, and the overall ecosystem; however, many of their competitors were still struggling with this. Moreover, in smaller countries XR vendors and consultants were seen to be not readily available, as XR was still seen to be a novel and niche market.

## Discussion and conclusion

**Fig. 1:**
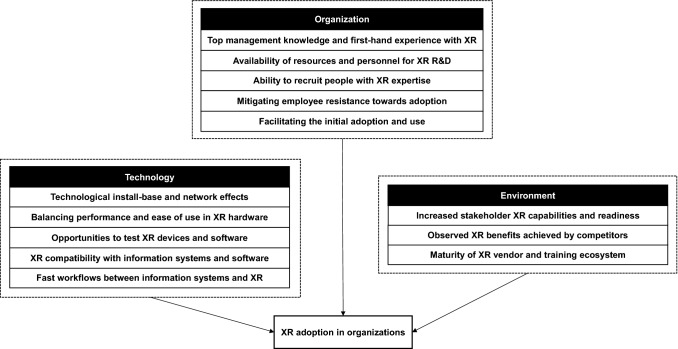
The identified XR adoption enabling factors categorized under the TOE framework

This mixed-methods study provides a holistic view on the current state of AR and VR adoption in European industrial companies and identifies key enabling factors affecting XR adoption. A cross-sectional online survey (*n* = 208) was carried out to answer the first research question: “*Do the current levels of XR awareness, use, and perceived limitations differ between European SMEs and larger companies?”* Our study revealed that, overall, there were no differences in perceptions or use levels between AR and VR. However, large companies positively differed from SMEs on AR use levels (*p* = 0.011, *p* < 0.05, *r* = 0.175) and more marginally with VR use levels (*p* = 0.06, *p* < 0.1, *r* = 0.13), even though there were no differences between SMEs and large companies regarding awareness or perceived limitations relating to AR and VR. In addition, we interviewed 45 companies and identified 13 enabling factors for XR adoption and categorized them under the TOE framework in order to answer the second research question: “*What are the critical enabling factors of XR adoption for SMEs?”* The summary of these findings is presented in Fig. 1. Further analysis presented in the next section found that eight of these enabling factors were specifically highlighted in the SME context.

### Theoretical contributions

The present study makes a twofold contribution to theory. First, the quantitative analysis based on an industry survey found there to be no statistically significant differences in organizations’ AR and VR use levels or the awareness and perceived limitations regarding these technologies. These results give further evidence and justification for examining both of these technologies simultaneously, as has already been practiced in prior literature (e.g., Davila Delgado et al. 2020; Steffen et al. 2019). These results also bring into question whether or not AR and VR really are at different development stages from an organizational point of view, as has been reported previously (e.g., Gartner 2017). Even though HMD implementations of AR are still likely less mature than VR implementations, it is possible that organizations view AR as a whole to be at the same level of maturity as VR when smartphone- and tablet-based AR are included. Moreover, analysis of the survey data found that larger companies were using AR and VR more than SMEs, although both were similarly aware of their potential and perceived similar levels of limitations in their adoption. This further corroborates earlier findings that large companies are more likely to adopt emerging technologies first before they have become well established in industry (Porter and Heppelmann 2017) and confirms the need to support, especially, SMEs in the adoption of digital technologies through the identification of enabling factors that are specifically applicable to them.

Second, this study provides an organizational perspective on XR adoption based on the TOE framework with a specific focus on SMEs. The present study contributes to the nascent literature on organizational XR adoption by uncovering key technological, organizational, and environmental enabling factors and assessing their specific importance for SMEs. The identified enabling factors and whether their importance is highlighted in the SME context are summarized in Table 6. Moreover, the novelty of the enabling factors is compared against previous findings from literature. This comparison provides a concise view on the state of extant XR adoption literature.

**Table 6:** Summary of XR adoption enabling factors

Enabling factor	Corroborating research	Highlighted in SMEs?	
		Yes/No	Description
*Technology*			
Technological install-base and network effects	–	Yes	SMEs often work in larger networks; proliferation of XR in other companies enables the use of XR in additional business processes
Balancing performance and ease of use in XR hardware	Berg and Vance (2017), Jalo et al. (2020), Masood and Egger (2020)	No	Both SMEs and large companies need to be able to choose appropriate equipment
Opportunities to test XR devices and software	Jalo et al. (2020)	Yes	SMEs often do not have the slack resources to experiment with XR hardware and software, which makes ease of testing more crucial for them
XR compatibility with information systems and software	Jalo et al. (2018, 2020), Masood and Egger (2019)	Yes	SMEs will likely not start tailoring XR solutions to their IS and software
Fast workflows between information systems and XR	–	No	Efficiency of workflows important for both SMEs and large companies
*Organization*			
Top management knowledge and first-hand experience with XR	Alam et al. (2021), Berg and Vance (2017) Chandra and Kumar (2018), Masood and Egger (2019) and Noghabaei et al. (2020)	No	Top management support for XR adoption critical in all companies
Availability of resources and personnel for XR research and development	Davila Delgado et al. (2020), Noghabaei et al. (2020)	Yes	SMEs often lack slack resources which could be directed toward XR implementations
Ability to recruit people with XR expertise	Badamasi et al. (2022), Davila Delgado et al. (2020)	Yes	Due to the novelty of XR, finding experts is difficult for most companies. However, unlike large companies, SMEs likely cannot afford specialized XR experts
Mitigating employee resistance toward adoption	Badamasi et al. (2022), Davila Delgado et al. (2020), Masood and Egger (2019)	No	Inertia and resistance possibly even higher in larger companies due to increased bureaucracy and organizational complexity
Facilitating the initial adoption and use	–	No	Positive first impressions with XR important for all companies
*Environment*			
Increased stakeholder XR capabilities and readiness	Alam et al. (2021), Chandra and Kumar (2018)	Yes	Critical for interorganizational business processes, SMEs likely do not have the leverage to require other companies in the network to acquire XR expertise
Observed XR benefits achieved by competitors	Alam et al. (2021)	Yes	SMEs often wait for larger and more risk-tolerant companies to demonstrate the effectiveness of new technologies
Maturity of XR vendor and training ecosystem	–	Yes	SMEs will likely need more external support for implementing XR

The identified technological enabling factors (Table 6) were found to be mainly focused on XR and IS compatibility, and in the diffusion of the technology in the larger ecosystem, which can enable the use of XR in external business processes due to increased network effects. As more organizations begin adopting XR, its value proposition for other organizations increases simultaneously, as it opens up new opportunities for collaboration. This is especially crucial for SMEs, as the intraorganizational application potential of XR will likely be wider in large companies. In addition, wider diffusion provides opportunities for organizations to test XR. This can be especially helpful for SMEs, which often do not possess the extra resources to obtain XR devices for experimentation purposes. Testing opportunities can also help companies in finding the right balance between performance and ease of use with their chosen XR solution. Last, although IS compatibility has been highlighted in extant literature (e.g., Davila Delgado et al. 2020), the rapidness of the IS-XR workflows is noted here as a distinct factor, as it can help in incorporating XR into everyday business processes. The off-the-shelf compatibility of XR with IS is also likely more relevant for SMEs, as tailoring of the solutions carries higher financial risks.

At the organizational level, the top management not only needs to be knowledgeable about XR (Berg and Vance 2017), but they also need to test these devices in practice due to their immersive and novel nature in order to grasp their enterprise-application potential. As SME managers can often also be the direct owners of the company, convincing them about XR’s potential can significantly help in securing the required resources for XR. Berg and Vance (2017) also noted that the VR champion in an organization should encourage the end users to test VR in practice to fully recognize its potential. Securing the needed personnel and development resources for XR was also found to be crucial, especially for SMEs that are often limited in this regard. The required XR expertise can be found by recruiting the necessary talent from external sources or internally from employees who have self-learned how to use XR. Although XR is still often perceived to be more applicable in entertainment rather than demanding industrial and engineering use (Davila Delgado et al. 2020), its hedonic use can also develop skills that can be applied in the organizational context. Mitigating employee resistance toward XR was also found to be critical, a theme that has also gained increasing interest in recent literature (see e.g., Kim and Kankanhalli 2009). Some of the most promising ways to mitigate such resistance include involving the employees in the XR development process from the beginning and ensuring the initial testing and use of XR to be as practical and engaging as possible. An organizational culture supporting innovation can also lower the threshold for experimentation.

The maturity level of the XR vendor and training ecosystem was found to be an especially relevant environmental factor for SMEs, which often do not possess sufficient capabilities for independent system implementation and training. Successful XR adoption by competitors can also create pressure for adopting XR. Such mimetic pressures were also found to be critical for the adoption of virtual worlds by Yoon and George (2013). However, our analysis also noted that XR adoption by competitors was likely to be more visible in external customer-facing processes, which are already more difficult to implement in comparison to XR utilization in internal business processes. Thus, if companies wait for visible signs of XR adoption in their competitors, they are likely lagging far behind them in applying XR, as XR will likely be initially adopted in internal business processes that then create organizational capabilities for wider XR use. At this point, the required XR capabilities and readiness of other stakeholders will also probably be higher.

In summary, although the affordances created by AR and VR can be slightly different (Steffen et al. 2019), common factors can be identified that are relevant for adopting both of these technologies, as they are essentially both focused on presenting digital information to organizational users visually in an immersive manner, and in enabling new ways to interact with this digital content. However, this study also shows that the importance of specific enabling factors can vary depending on the size of the company and its business environment.

### Practical contributions

From a practical point of view, the holistic multi-country overview provided by this study highlights key issues for industry managers aiming to invest in XR by highlighting critical technological, organizational, and environmental factors they need to focus on to ensure a smoother adoption process. The in-depth analysis of enablers based on the three dimensions of the TOE framework can represent a reference for organizational managers and decision-makers interested in identifying existing barriers in their companies and in leveraging the most relevant enablers to drive their companies toward effective adoption of XR. In particular, aside from the technological and organizational aspects, the multidimensional level of analysis includes essential environmental factors such as the maturity of the related innovation ecosystem, which can provide essential support for organizations considering adopting XR. The findings of the study can thus help companies in systematically addressing the key issues which can hinder organizational XR adoption.

SME managers and decision-makers can especially benefit from understanding which XR adoption factors are highlighted in their own context, thus enabling them to shape their digital transformation path according to the specific competencies, resources, and strategic goals pursued by their organization. As SMEs often do not have enough slack resources for experimenting with new technologies on their own, seeking external opportunities for testing XR solutions and acquiring expertise from the external innovation ecosystem can thus be specifically useful for SMEs. These trial opportunities can also help them choose appropriate XR equipment and enable their top management to test XR in practice to help them better understand how XR might fit in with their current IS and software, as well as their overall business strategy. As many SMEs often follow larger companies when it comes to adopting new technologies, the survey overview about the XR adoption situation in European companies can also help managers in evaluating the overall market situation and in determining whether their companies should start investing in XR. Monitoring how widely XR has already diffused within their stakeholders and what level of capabilities they possess can also help companies understand in which business processes XR can already be leveraged effectively.

### Limitations and future research

This study has certain limitations pertaining to its quantitative and qualitative aspects. First, the survey data collection was placed at the beginning of the COVID-19 pandemic, which was a turbulent period for many companies. This may have influenced what types of companies were able to answer the survey. The shift toward remote work has also possibly spurred further interest in XR. Longitudinal studies on organizations’ perceptions and situations after the pandemic might provide different results. As the pace of digitalization and adoption of technologies has increased more generally (Denning and Lewis 2020) as well as specifically due to the pandemic (OECD 2021), more research is needed on what company traits and capabilities are highlighted in the effective adoption of emerging technologies, both in SMEs and large companies. Moreover, even though both the quantitative and qualitative samples were mainly focused on SMEs, larger companies were also included. However, we view this choice as justified, as this sample is more representative of the overall enterprise market composition of the European industrial sector. This also allowed us to compare whether the situation with AR and VR differed between SMEs and larger companies and to assess the specific importance for SMEs of key technological, organizational, and environmental enabling factors identified in this research.

Second, although the semi-structured interview protocol was shared among the researchers and iteratively refined based on their feedback, it is possible that both the interviewers and the interviewees understood and interpreted the questions differently due to cultural and person-specific issues. Moreover, neither the relative significance nor the interrelationships of the identified enabling factors were examined in this study. Future research could thus operationalize the enabling factors and quantitatively evaluate their importance for organizations. Our findings are also mainly focused on the organizational level of adoption. As XR can be used to radically transform organizational activities and social structures, more research on employee perspectives on potential conflicts and changes that XR adoption can bring about could prove to be useful. Both quantitative and qualitative longitudinal pre- and post-adoption research designs could be employed to examine these issues.

## Data Availability

The fully anonymized data of this study are available from the corresponding author on reasonable request.

## Appendix

### Interview protocol

The interview protocol below was phrased to be used with companies who have not yet used AR or VR. Another interview protocol was also developed to be used with companies who were already using AR or VR. The protocol had only slightly differed phrasings, so it was omitted here due to space limitations.

### Familiarity with AR/VR

Shortly, how aware are you of Augmented Reality (AR) or Virtual Reality (VR)?

- Have you used them yourself? Or have you seen them being used somewhere?

Has your organization thought about using AR and VR? Which one has more potential for your organization?

- Where and how could you use them in your organization?
  - e.g., visualizations, information access, multi-user collaboration, remote support?
  - Which tasks and processes?
- Has your organization been testing any kind of AR or VR devices?
  - Do you know where you could test them?
- Why haven’t you started using AR or VR yet?
  - Is the problem with the technology itself (e.g. cost, too complex…)?
  - Or are there some organizational barriers that prevent you from using AR or VR (e.g. ease of integration with business processes)?
  - Or is the issue with the employees (e.g. lack of skills)?

### Organizational issues

How knowledgeable is top management about AR or VR?

- Is someone in top management promoting their use?

Do you think your organization could start using AR or VR on your own (e.g., buy devices, install software, teach your employees on how to use the technology) or would you need external support?

- In what areas would you need support?
  - e.g. technological issues, adapting business processes, training employees to use the technology…
- Where would you want to get the support from?
  - University collaboration? Industry associations? Technology vendors?

What kind of skills would your employees and managers need to learn to use AR or VR effectively?

- Do you think your employees could learn to use AR or VR by themselves?
- Where do you think these skills could be learned?
  - Self-learning? Internal company courses? Vendor training? Consultant companies?

How could universities help your organization in the adoption of these technologies?

- What sort of cooperation would you prefer?
- What would increase your interest in adopting these technologies?

How well does your organizational culture support experimenting and testing new technologies?

### Technological issues

What kind of content would you want to use in AR or VR (e.g. 3D models, visualizations, organizational data etc.)?

- How would you get this content to AR or VR?
- What kind of issues do you think you would face in integrating AR or VR into these systems?

Have you been able to test out different AR or VR solutions?

- Where did you test them? How was the experience?
  What benefits do you think AR or VR would bring to your organization?
  Do you think your employees would resist using AR or VR? Why?
  Do you plan to use AR or VR in the future?
- When?
- What needs to happen with these technologies for you to start using them in your organization?

### External issues

Have your competitors used and benefited from AR or VR?

- Is this creating pressure to adopt these technologies?
  Are any of your stakeholders (e.g. suppliers, customers) using these technologies?
- Is this creating pressure to adopt these technologies?
